# COVID-19 Safety Measures in the Food Service Sector: Consumers’ Attitudes and Transparency Perceptions at Three Different Stages of the Pandemic

**DOI:** 10.3390/foods11060810

**Published:** 2022-03-11

**Authors:** Heidi Vandenhaute, Xavier Gellynck, Hans De Steur

**Affiliations:** Division of Agri-Food Marketing & Chain Management, Department of Agricultural Economics, Ghent University, Coupure Links 653, 9000 Ghent, Belgium; heidi.vandenhaute@ugent.be (H.V.); xavier.gellynck@ugent.be (X.G.)

**Keywords:** COVID-19, Belgium, consumer behaviour, food service sector, safety measures, transparency

## Abstract

The food service sector was among the hardest hit by the COVID-19 pandemic. This study aims to examine consumers’ attitudes towards and transparency perceptions of COVID-19-related safety measures and to identify determinants of consumers’ intentions and behaviour regarding visiting restaurants and bars once reopened. By also surveying food service businesses, this study allows for comparison between both target groups. A total of 1697 consumers and 780 businesses participated in this study, conducted in Belgium both during and in between waves of infections. The findings demonstrate that consumers evaluated safety measures as important when revisiting restaurants and bars, against business owners’ expectations. Both consumers’ revisit intentions and behaviours are influenced by the perceived importance of hygiene measures (negatively) and past visit frequency (positively). This study highlights the importance of good compliance with safety measures as a strategy to attract customers during the reopening period. Further, our findings emphasize the importance of transparent communication by food service businesses and the government.

## 1. Introduction

The COVID-19 (COrona VIrus Disease-2019) pandemic left behind a trail of unprecedented consequences on everyday life worldwide. Over 9.8 billion vaccine doses have already been administered [1] and enabled many countries to gradually return to normalcy. However, with the arrival of new variants, the pandemic remains pervasive with over 323.6 million reported cases and 5.5 million confirmed deaths as of 16 January 2022 [2]. To slow down the spread of the virus in order to protect their healthcare systems from overloading, governments worldwide implemented strict health measures, imposing major restrictions on all aspects of daily life. Social distancing measures have been prioritized in many countries and this has necessitated the closure of non-essential services, including schools, offices, restaurants, and hotels [3,4]. To reduce interactions and, thus, virus transmission between people, public gatherings were banned, teleworking became the norm and travelling was suspended [5,6]. As the number of positive cases, hospitalisations, and deaths slowly decreased, mitigation measures were relaxed or lifted, and social bubbles were expanded. Nevertheless, many European countries faced a second, third and even fourth wave of infections, along with waves of gradually tightening and relaxing original measures.

Disease outbreaks have the potential to disrupt existing food systems and create food crises [7,8]. Due to the COVID-19 pandemic, food supply chains were confronted with demand shocks, including changing purchasing and consumption patterns [9,10]. Over the last decades, an increasing number of people depend significantly on out-of-home eating services for their daily dietary intake [11,12]. With the closure of restaurant, bars, schools, and offices, meals prepared and consumed at home have replaced the food service sector, exerting additional pressure on the food retailing sector [9]. While out-of-home food consumers are forced to adopt a less convenient lifestyle, catering services see both their revenues and chances of survival either drop or disappear.

## 2. Literature Review and Aims

### 2.1. COVID-19 and (Out-Of-Home) Food Consumption Behaviour

Although it is clear that COVID-19 has impacted food consumption behaviours, preliminary results are diverse and contradictory. Shifts towards both healthier and unhealthier diets were identified during the COVID-19 pandemic and lockdowns. On the one hand, lockdown restrictions led to the adoption of a healthier diet and a reduced consumption of unhealthy foods [10,13]. On the other hand, an increase in ‘comfort food’ consumption and snacking was reported, leading to a decrease in dietary nutritional quality [14,15,16]. Several reasons may account for these mixed effects. A balanced and diversified diet can help in maintaining and strengthening immunity, which is essential when dealing with viral threats [13,17]. Moreover, with the closure of restaurants, out-of-home eating was mostly substituted by home-cooked meals, which is typically considered to be a healthier choice [18]. As out-of-home food consumption is associated with a higher intake of energy and fat [11,19], people who used to have more meals out-of-home before the lockdown showed an increased adherence to healthier dietary habits during the lockdown [13]. The increased intake of unhealthy food products can be attributed to panic buying and negative emotions, such as anxiety, stress, and boredom, related to the pandemic and the consequential confinement [10,14,20].

Research on the impact of the COVID-19 pandemic on food purchase behaviour, and takeaway and meal delivery in particular, provides inconclusive results. During lockdown periods, the use of food delivery and takeaway was found to both decrease [10,21,22] and increase [23]. Although there is currently no evidence of COVID-19 transmission through food and food packaging [24], fear for unnecessary exposure might explain why people reduced their frequency of ordering food from restaurants [10]. Perceived risks related to COVID-19 negatively affected the intention to buy food through online food delivery services [25,26]. In contrast, purchase intentions during lockdown were positively influenced by the frequency of online food ordering before COVID-19 [26]. According to Poelman, et al. [23], 30% of those who previously used meal delivery services, did so more frequently during lockdown, especially for meals from local restaurants, with a bias towards highly educated and young consumers. As a higher education level is also associated with eating out-of-home [11,27], the increased use of meal delivery and takeaway might be seen as a means to recreate the restaurant experience at home [23].

It is largely unclear how people’s consumption and purchasing behaviour evolved as lockdown restrictions were lifted and countries moved towards a ‘new normal’. Undoubtedly, COVID-19 will have changed consumers’ out-of-home food consumption behaviour in the short and long term, but research focusing on this topic is still scarce [28]. As dining out implies a setting that involves a large number of people in close proximity to one another for an extended period of time, human interaction, and thus risk of infection, is inherent in visiting food service businesses [28]. Further, high-touch surfaces outside the food preparation areas, e.g., restaurant menus, represent a potential risk of cross-contamination [29,30]. Therefore, many consumers did not feel comfortable and were reluctant to revisit restaurants and bars upon reopening [31,32,33]. In the United States, for instance, Gursoy and Chi [31] found that more than half of consumers were not willing to revisit food service businesses immediately and of those who already had the opportunity to return, only one in four did. This is consistent with the study by Taylor [32], where 50% of respondents had dined in at a restaurant three weeks after lockdown restrictions were lifted across the US. Consumers’ intentions towards dining out were negatively affected by a high-risk perception of COVID-19. The more people are concerned about eating out-of-home, the more likely they are to avoid it [28], while trust in the ability of restaurants and bars to handle COVID-19 positively affects consumers’ intention to visit them during the pandemic [34].

### 2.2. Safety Measures in the Food Service Sector

As it becomes clear that not everyone will be rushing back to restaurants and bars in the short term, it is of utmost importance that business owners do everything they can to improve consumers’ willingness to revisit them. Many countries have published policy documents with regulations for the safe reopening of food service businesses [35]. Safety measures in restaurants and bars reduce the possibility of infection and consumers’ perceived risk. By thus ensuring customers’ health, customers are motivated to dine out, indicating the importance of safety measures [28,34]. Key safety measures customers expect from food service businesses include visible sanitation efforts, social distancing, limited number of customers, more thorough and frequent cleaning of high-touch surfaces and employee training of health and safety protocols [31].

Social distancing and safety measures can be implemented in different ways, which may elicit different attitudes towards dining out. Research has indicated that consumers prefer restaurants that use partitions to ensure social distancing between different parties [32]. Partitioned restaurants, where physical barriers create individual spaces within a larger room, were considered safer, cleaner, and more sanitary. Similarly, perceived threat of COVID-19 increased preferences for restaurant set-ups with private dining tables or rooms [33]. Both cleanliness and customers’ cleanliness perceptions have become increasingly important since the COVID-19 outbreak [30,32]. Perceived restaurant cleanliness has a positive effect on customers’ satisfaction, which in turn positively impacts revisit intention [32,36,37], whereas cleanliness of restaurants is a key determinant of consumers’ decision to select or return to a restaurant [38,39]. By prioritizing cleanliness, food service businesses may not only ensure the health of customers and employees but also attract customers by meeting their cleanliness expectations [30,32]. Adequate implementation as well as communication of the measures were considered important for consumers to enjoy eating out with lower perceived risk of becoming infected [28,34]. As part of their recovery strategy in response to SARS, restaurants also used cleanliness as a selling proposition [40]. By communicating the safety measures taken to ensure customers’ health, perceived risk of dining out is expected to decrease, while customers will feel confident to come back [40].

Food service businesses, among the hardest hit by the COVID-19 pandemic, are confronted with numerous uncertainties while facing difficult circumstances. As lockdown periods of mandatory closure (except for takeaway or delivery) were alternated with periods of reopening under strict conditions, restaurants and bars had to drastically change the way they operate to ensure compliance with the imposed safety and social distancing guidelines. Doing so safeguards the health of both customers and employees and encourages customers to revisit their businesses [31]. However, after having endured months of closure, such safety measures further challenge companies’ chances of survival. The enforced measures result in additional expenses, e.g., for disinfecting and protective materials, as well as reduced revenues, e.g., due to limited capacity and imposed curfew. Revenues are further diminished by consumers’ reduced demand for restaurant services due to the risk involved and their avoidance of eating out [28,41].

### 2.3. Transparency of Government and Businesses’ Communications

A government’s control strategies can only be considered successful when its measures are broadly accepted. Non-compliance of safety measures renders them ineffective. Trust in government is key for effective implementation of policy measures that rely on behaviour [42,43]. Previous studies have demonstrated a positive correlation between adoption of recommended health precautions and trust in authorities. People are more likely to comply with health-related recommendations when they trust their efficacy and the institutions issuing them, along with the latter’s competence to contain the pandemic [42,43,44,45,46,47,48]. Moreover, the adoption of policy recommendations is related to the communication strategy used. People are more likely to undertake recommended precautionary behaviours if the information communicated by the authorities is perceived clear, consistent, sufficient and helpful [46]. Policy communications should be credible and coherent; measures that are inconsistent, unclear, and open to interpretation will cause confusion and undermine compliance [44,49]. A good level of understanding of the measures and the rationale behind them is positively correlated with higher acceptance and more compliant behaviour [47,49].

In addition, clear and unambiguous health information are considered essential in maintaining trust in authorities. A policy of open and transparent communication, providing all necessary information, leads to more public trust [50]. A positive relationship exists between perceived government transparency and trust in government [51]. The same applies at the level of businesses; the food service sector can rebuild consumers’ trust by being transparent, appearing credible and sharing timely, accurate, consistent and reliable information [34,52]. Yost and Cheng [53] suggest the importance of restaurant transparency to regain consumers’ trust, which may motivate them to resume dining out during the pandemic. Trust in government is also positively associated with the willingness to engage in prosocial behaviours, e.g., making donations to help those who suffer from the pandemic [43]. Further, solidarity with the food service sector, i.e., visiting bars and restaurants or using online food service applications to protect them from bankruptcy, unemployment and liquidity shortage, influences consumers’ visit intention during the COVID-19 pandemic [34,54].

### 2.4. Aims

It is unclear to what extent food service businesses will (have to) change and adapt to the new reality and how this relates to their customers’ attitudes towards safety when dining out. More research is needed to assess how safety measures will influence consumers’ dining out intentions and behaviours. For food service businesses, this is crucial to restore the demand for their services that is required to survive and recover from this crisis. Therefore, this study examines attitudes, perceptions, and behaviour from the perspective of both consumers and businesses. At the consumer level, this study aims to evaluate their attitudes towards expected and imposed safety measures and to gain insight into consumers’ decisions to either visit restaurants and bars as soon as they reopen or postpone their visits. In addition, by investigating food service businesses’ perceptions, this study allows for comparison between both target groups. As such, this study attempts to answer the following research questions: (RQ1a) how important do consumers consider safety measures when revisiting food service businesses?; (RQ1b) how does this relate to businesses’ perceptions of safety measures and expectations of their customers’ attitudes?; and (RQ2) what are the determinants of consumers’ intentions and behaviour regarding out-of-home consumption in (post) pandemic times? For RQ2, following prior research findings, we hypothesised that consumers’ attitudes towards sanitation negatively influence revisit intention and behaviour, whereas their past visit frequency has a positive influence.

Furthermore, it is highly relevant to explore how perceptions of transparency of COVID-19 (safety) measures link with attitudes and behaviour towards them and what solidarity intentions and expectations consumers and businesses respectively have. Therefore, this study also investigates the following research questions: (RQ3a) to what extent are consumers willing to financially support food service businesses?; (RQ3b) how does this relate to businesses’ expectations of their customers’ willingness?; and (RQ4) to what extent do consumers’ transparency perceptions of communications by food service businesses and the government correlate with their attitudes? In relation to RQ4, we hypothesised that perceived business and government transparency is positively correlated with consumers’ perceptions when dining out and acceptance of policy decisions, respectively.

## 3. Materials and Methods

### 3.1. Survey Design

Three different cross-sectional studies were conducted at three different stages of the COVID-19 pandemic. All studies addressed both consumers and the food service sector (restaurants and bars). Three standardized surveys were developed, one per study, and were each divided into two sub-surveys for the different target groups. Although the studies were targeting different stages and measures during the pandemic, all surveys were structured in a similar way.

The consumer questionnaires consisted of three parts. The first part contained behavioural questions regarding out-of-home food consumption and takeaway, before and since COVID-19. Past visit (study 1, 2) and takeaway (study 3) frequencies were measured by recoding a 10-point scale, ranging from 1—‘never’ to 10—‘daily’, into frequencies per week. Consumers’ intentions to revisit food service businesses were evaluated in the first study, while the second study assessed consumers’ revisit behaviours. Revisit intention and behaviour were both recoded into dummy variables (yes/no).

The second part measured attitudes towards and perceived transparency of (safety) measures and decisions issued by the government. Safety measures, aimed at preventing virus transmission during food service visits, were developed through consultation of experts (study 1) and government documents (study 2) [55] in light of the current policy. The measures included items such as “Service is performed with mouth mask”; “No possibility of self-service or buffet” and “Clients can only consume while seated”. Attitudes were evaluated based on 5-point importance scales, with values ranging from 1—“not at all important” to 5—“very important”. While the first study dealt with 21 safety measures expected to be imposed when food service businesses reopen, the second study focused on 14 actually imposed safety measures, extended with perceived compliance of the measures and perceived safety when revisiting. Imposed measures were slightly different from what was expected a priori. This discrepancy was a result of the growing body of knowledge on the virus and the continuously evolving epidemic situation. Perceived compliance refers to the extent to which bars and restaurants adhere to imposed safety measures, while perceived safety relates to the extent to which consumers felt safe during their visit. Both variables were measured using a self-constructed item on a 5-point Likert scale. The third study focused on consumers’ attitudes towards government decisions (5-point Likert scale, “I support the government’s decision to close food service businesses”) and on consumers’ willingness to financially support the sector. Five support actions were evaluated, namely: “Extra use of takeaway/delivery of meals”; “Extra tip when using takeaway/delivery of meals”; “Purchasing vouchers”; “Support crowdfunding campaign” and “Paying extra corona contribution at next visit”, on a 5-point willingness scale.

Perceived transparency regarding businesses’ (study 2) and government (study 3) communication was assessed by using items from Rawlins [56] and measured on a 5-point Likert scale. The subscale substantial information (7 items, namely: “The information communicated by the business/government about the measures is timely; relevant; consistent; complete; easy to understand; accurate; reliable.”) was supplemented by one self-constructed item (“The information communicated by the business/government about the measures explains the rationale.”). The questionnaires concluded with a set of profiling variables related to socio-demographic characteristics, such as age, gender, and education level.

The surveys addressing food service business owners were composed of two parts. The first part measured attitudes towards government safety measures, reviewed by experts, on 5-point scales. Businesses evaluated the same set of safety measures as consumers, both expected (study 1) and imposed (study 2), though with the option to indicate “not applicable to my business”. The first study assessed businesses’ expectations of their customers’ attitudes towards the measures (5-point importance scale), while the second study focused on the perceived impact of the measures on businesses’ profitability, using a scale ranging from 1—“not at all” to 5—“very much”. Businesses’ expectations of their customers’ willingness to make a personal contribution through the five aforementioned support actions were evaluated in the third study (5-point willingness scale). The surveys concluded with profiling questions regarding business type in order to distinguish between food service businesses serving food and drinks (restaurant) and those only serving drinks (bar).

### 3.2. Data Collection

Data were collected in May 2020 (study 1), June 2020 (study 2) and November 2020 (study 3) through online surveys. The first and third study were conducted during the first and second wave of COVID-19 infections respectively, with food service businesses being mandatory closed. Data collection for the second study was performed in between waves of infections, when reopening was allowed. In order to facilitate data collection, questionnaires were integrated into Qualtrics software for both stakeholder groups. By using a convenience sampling procedure, the surveys were administered to the target groups, both food service businesses and food service customers. Stakeholders from sector organisations distributed the survey among their members, while social media channels were used to disseminate the survey to the general public. Regarding food service businesses, gourmet restaurants, bistros/brasseries, fast food restaurants, buffet restaurants and bars, whether or not serving food, were targeted. Food service customers had to eat or drink out at least once a year to be included. For both surveys, participation was further restricted to people with a minimum age of 18. After removing incomplete responses, the final sample consisted of 1083, 309 and 305 consumers and 306, 221 and 253 businesses for study 1, 2 and 3, respectively. The higher response rate for the first consumer study can be attributed to the strict lockdown in place at the time of survey administration.

Flanders (Belgium) was targeted as study location. Belgium reported its first confirmed case of COVID-19 on 4 February 2020 and first death on 10 March 2020. From 14 March 2020 onwards, restaurants and bars had to close their businesses, only to be allowed to reopen on 8 June 2020. Mandatory closure of dine-in services during lockdown periods caused restaurants to set up takeaway and delivery services. Reopening opportunities were accompanied with strict safety measures designed to prevent the spread of the virus and protect the health of both employees and customers. Belgium successfully flattened the epidemic curve in April 2020, yet the country experienced its second wave of COVID-19 in the final months of 2020. The government decided to reclose restaurants and bars, starting from 19 October 2020 onwards, initially for four weeks, but ultimately for over six months, as a response to the third wave. Meanwhile, several financial support measures and actions were introduced by the Belgian federal government and the public respectively to ensure the survival of these businesses. Further, similar to many countries, the consumption pattern of out-of-home eating to satisfy daily dietary needs has over time gained prominence in Belgium [57,58], illustrating the relevance of Flanders as a study location. Table 1 presents an overview of the survey development and data collection process.

### 3.3. Data Analysis

Statistical analyses were performed using IBM SPSS Statistics 27. Principal component analysis (PCA) with Varimax rotation was used to explore the underlying structure both of consumers’ attitudes towards the expected to be imposed (study 1) and imposed (study 2) safety measures on food service businesses and of consumers’ transparency perceptions regarding government communication (study 3). The Kaiser–Meyer–Olkin (KMO) measure of sampling adequacy and the Bartlett’s test of sphericity provided acceptable values indicating the meaningfulness of performing a factor analysis on the chosen variables. The latent root criterion was applied so to only retain factors with an eigenvalue above one [59]. Factor loadings above 0.5 were considered practically significant, following the rule of thumb of Hair, et al. [59]; items with factor loadings below 0.5 were omitted. Since businesses evaluated the same set of safety measures, the factorial structure from the consumer oriented PCA was used to group businesses’ attitudes, allowing for a comparison of food service businesses and consumers. Internal consistency of the factors was tested through McDonald’s omega in order to justify the creation of composite variables based on the average score on the underlying items of each factor.

Hierarchical binary logistic regressions (enter method) were performed to estimate the role of the aforementioned factors, past frequency of out-of-home food consumption and socio-demographic variables (age, gender, education level) on consumers’ revisit intention (0—postpone visit; 1—retake visit; study 1) as well as revisit behaviour (0—visit postponed; 1—visit retaken; study 2). Outliers, i.e., cases with standardized residuals above |2| [59], were listed, subjected to visual inspection of DFBeta and stepwise removed (<3% of cases) until a suitable model was achieved. For all estimated models, no indications of major problems with multicollinearity were apparent. Although positive bivariate correlations were found between established factors, the coefficients were below the threshold (0.7), while collinearity diagnostics showed VIF (variance inflation factor) values well below 10 [59,60]. Different goodness-of-fit statistics were calculated to estimate model fit. The significance of the likelihood ratio chi-square tests indicated that the models containing the independent variables represented a significant improvement in fit relative to the model without variables (‘null’ model). Nagelkerke R^2^ values, reflecting the amount of variation accounted for by the logistic model, showed an improvement from Model 2 relative to Model 1.

Other data analysis techniques used included descriptive, univariate, and bivariate analyses. Differences in socio-demographics between samples were tested with one-way ANOVA and chi-square tests. Differences in factor means (study 1, 2) were tested with paired samples *t*-tests, while independent samples *t*-tests were used for differences in composite variables (derived from the factors; study 1, 2) and (expectations of) willingness to support (study 3) between the target groups. Differences in takeaway consumption frequency (study 3) were tested with both paired samples *t*-tests, independent samples *t*-tests and bivariate correlations. Bivariate correlations were also used to examine associations between perceived transparency and other variables (study 2, 3).

## 4. Results

### 4.1. Sample Descriptives

The total sample consisted of 1697 consumers and 780 food service businesses. Table 2 presents the characteristics of the sample of each study. There were no differences between the consumer samples in terms of age (one-way ANOVA; *F* = 2.47; *p* = 0.085) and gender (chi-square test; 𝜒^2^ = 3.63; *p* = 0.163). The average age of the sampled consumers was between 42 and 44. Both female and higher educated people are slightly overrepresented in the samples. At the level of food service businesses, business type was equally distributed between samples (chi-square test; 𝜒^2^ = 4.68; *p* = 0.096). Roughly three out of four sampled businesses were restaurants serving both food and drinks, while bars that only serve drinks were less represented.

### 4.2. Expected Safety Measures in Pandemic Times (Study 1)

#### 4.2.1. Consumers’ Attitudes and Businesses’ Expectations of Their Customers’ Attitudes

PCA was performed to explore the underlying structure of consumers’ attitudes towards 21 expected safety measures and resulted in a factorial structure with three factors. Three items were stepwise excluded (loadings < 0.5), while 18 items were retained, all loading well on one of the three factors. The results of the final factor analysis with 18 items are summarized in Table 3.

Factor 1 represents hygiene measures, which particularly focus on disinfecting hands and surfaces. Factor 2 deals with measures aimed at avoiding the sharing of objects between customers, such as menus and salt shakers. Factor 3 includes organisational measures that assure a well-organised flow of clients in the establishment. McDonald’s omega values for the three factors indicated good internal consistency and allowed for the development of composite variables for factor 1 (seven items), factor 2 (five items) and factor 3 (six items). Table 3 also presents item and factor means. Paired samples *t*-tests indicated significant differences between the factor means. The mean of factor 1 (hygiene; $\bar{x}$ = 3.82 ) is significantly higher compared to factor 2 (avoidance;$\bar{x}$ = 3.70) (*t* = 5.73; *p* < 0.001), which in turn has a significantly higher mean than factor 3 (organisation;$\bar{x}$ = 3.36) (*t* = 14.58; *p* < 0.001). While all items and factors are considered important when revisiting food service businesses, sanitary measures appear to be the priority for consumers.

**Figure 1:**
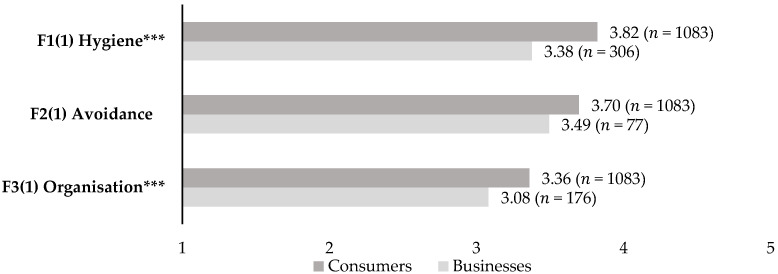
Expected safety measures: consumers’ attitudes vs. businesses’ expectations of their customers’ attitudes. Note: not all statements were evaluated by all business owners due to irrelevance, explaining the differences in *n* between factors; *** *p* < 0.001; FX(Y) with X = number of factor, Y = number of study.

To allow for comparison with restaurants’ and bars’ expectations of their customers’ attitudes towards safety measures in food service businesses, the same factors were developed. McDonald’s omega values above 0.7 justified the calculation of composite variables of hygiene and avoidance measures at the level of businesses. Internal consistency for the organisational measures was lower (McDonald’s omega = 0.667), yet close to the threshold of 0.7 and still acceptable for exploratory research [59]. Surprisingly, businesses’ expectations of their customers’ attitudes towards safety measures in their businesses are significantly different from consumers’ stated attitudes (Figure 1). Independent samples *t*-tests indicated significantly lower mean scores for hygiene (*t* = 8.71; *p* < 0.001) and organisational measures (*t* = 3.91; *p* < 0.001) from the businesses’ perspective compared to the consumers’ perspective.

#### 4.2.2. Determinants of Consumers’ Revisit Intentions (Study 1)

The first study revealed that 58.2% of respondents had the intention to immediately revisit food service businesses once reopened. To understand which factors influenced consumers’ intentions to either postpone or retake visits when restaurants and bars reopen, a binary logistic regression model was estimated. A hierarchical approach was used to assess the associations between attitudes (Model 2) and intention, controlling for socio-demographics and past behaviour (Model 1). Thirty outliers were omitted, yielding a total of 1053 valid responses. The results are summarized in Table 4. The significance of the likelihood ratio chi-square tests and the Nagelkerke R^2^ values (0.125 and 0.279 for Models 1 and 2 respectively) indicated a moderate model fit.

The first model (Model 1, one block) indicates that the probability of having the intention to revisit restaurants and bars was positively influenced by respondents’ visit frequency before the lockdown. An increase in the frequency of out-of-home consumption by one visit per week increased the odds of intending to revisit by 40%. Furthermore, gender had an effect on revisit intention; being male increased the odds of intending to revisit (odds ratio: 1.63). Age and education did not significantly affect revisit intention. From the final model (Model 2, two blocks), attitudes towards safety measures related to food service businesses were identified as significant determinants of consumers’ revisit intention; the importance of hygiene, avoidance and organisational measures had a significant negative influence. A one-unit increase in the attitude score decreased the odds of revisit intention by a factor in the range of 0.60 to 0.64. Respondents who valued the safety measures more were less likely to plan to retake their visits immediately and rather intended to postpone, with the risk of contamination as the major reason to do so (65%). This demonstrates the importance for businesses to strictly adhere to imposed measures in order to persuade customers to revisit their establishments during the COVID-19 pandemic. The effect of gender decreased in Model 2 and was no longer significant.

### 4.3. Imposed Safety Measures in Pandemic Times (Study 2)

#### 4.3.1. Consumers’ Attitudes and Perceived Impact on Businesses’ Profitability

PCA was performed to explore the underlying structure of 14 actual imposed safety measures. A two-factor solution was recognized, with factors conceptually similar to the factors ‘hygiene’ and ‘organisation’ identified in study 1. Here, the factor ‘avoidance’ was not identified, and items related to avoid the sharing of objects between customers loaded on other factors. To compare both studies, all three items related to ‘avoidance’ were removed in this analysis. This yielded high factor loadings for the remaining 11 items. The results are summarized in Table 5.

Similar to study 1, factor 1 deals with hygiene measures and factor 2 is clearly linked to organisational measures. McDonald’s omega values for the two factors indicated good internal consistency and allowed calculations of composite variables for factor 1 (seven items) and factor 2 (four items). Table 5 also presents item and factor means, as derived from attitude scores on a 5-point importance scale. Paired samples *t*-test indicated significant differences between factor means (*t* = 14.84; *p* < 0.001), with hygiene measures ($\bar{x}$ = 4.27) considered to be more important than organisational measures ($\bar{x}$ = 3.66).

**Figure 2:**
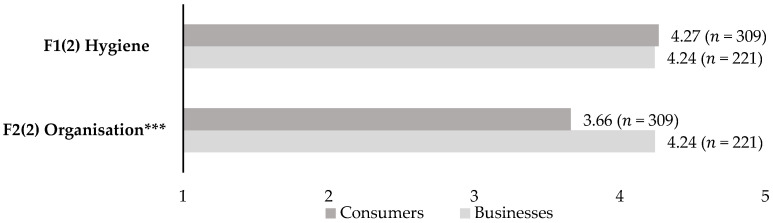
Imposed safety measures: consumers’ attitudes vs. perceived impact on businesses’ profitability. Note: *** *p* < 0.001; FX(Y) with X = number of factor, Y = number of study.

The factors grouping hygiene and organisational measures were also used to compare consumers’ attitudes towards the measures with the perceived impact of the measures on the profitability of restaurants and bars. As for the latter, composite variables were calculated (McDonald’s omega > 0.7). It becomes clear that both hygiene and organisational measures have a large perceived impact on businesses’ profitability. Independent samples *t*-tests indicated significantly lower mean scores for organisational measures (*t* = 7.17; *p* < 0.001) from consumers’ perspectives (importance) compared to businesses’ perspectives (perceived impact on profitability) (Figure 2).

#### 4.3.2. Determinants of Consumers’ Revisit Behaviour (Study 2)

In the second study, 69.3% of respondents indicated that they immediately revisited food service businesses as soon as they were allowed. To understand which factors influence consumers’ actual behaviour to either postpone or retake visits to restaurants and bars since reopening, another binary logistic regression model was estimated. A hierarchical approach was used to assess the associations between attitudes (Model 2) and current behaviour, controlling for socio-demographics and past behaviour (Model 1). Eight outliers were omitted, yielding a total of 301 valid responses. The results are summarized in Table 6. Certain goodness-of-fit statistics (likelihood ratio, Nagelkerke R^2^ of 0.289 and 0.348 for Models 1 and 2 respectively) were calculated and indicated moderate to good model fit.

The first model (Model 1, one block) indicates that the probability of revisiting restaurants and bars was positively influenced by respondents’ visit frequency before the lockdown. Increasing the frequency of out-of-home consumption by one visit per week, increased the odds of revisiting by a factor of 2.63. Moreover, age and education had an impact on revisit behaviour. A 10-year increase in age was associated with an 28% decrease in the probability of revisiting; higher educated people were less likely to retake visits immediately (odds ratio: 0.36). When looking at the complete model (Model 2, two blocks), attitudes towards hygiene measures were identified as another significant determinant of consumers’ revisit behaviour; a one-unit increase in the attitude score decreased the odds of revisiting by a factor of 0.44. The more respondents value the hygiene measures, the less likely they are to retake their visits immediately, hence more likely to postpone. The attitudinal variable related to organisational measures as well as gender did not significantly affect revisit behaviour.

### 4.4. Post-Pandemic Behaviour and Willingness to Support (Study 3)

Mandatory closure of dine-in services during lockdown periods led to a significant increase in consumers’ ordering frequency of takeaway meals (paired samples *t*-test; t=9.35; p<0.001). Whereas before the COVID-19 pandemic people chose takeaway on average once per month (0.21 times/week, S.D. = 0.33), this doubled during lockdown periods (0.45 times/week, S.D. = 0.51). However, consumers expected to reinstate their pre-pandemic behaviour in terms of takeaway and out-of-home consumption once the pandemic was over. No significant differences in lockdown takeaway consumption were found for gender and education level, nor was there a correlation with age.

**Figure 3:**
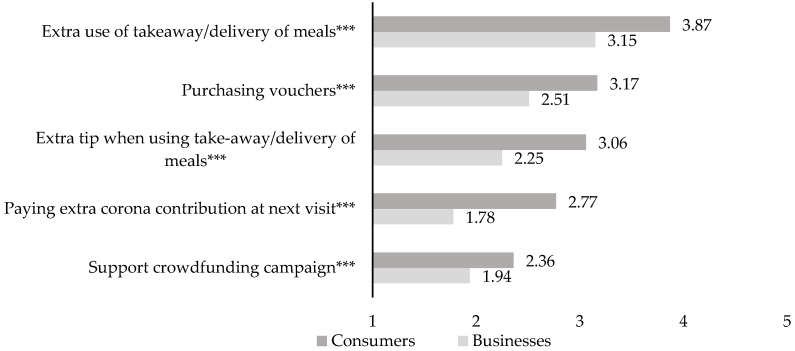
Consumers’ willingness to personally contribute (n=305) and businesses’ expectations of their customers’ willingness (n=253). Note: *** p<0.001.

Figure 3 shows consumers’ willingness to contribute versus businesses’ expectations of their customers’ willingness for five different support actions. While consumers and businesses ranked the options nearly identical, independent samples *t*-tests revealed significant differences between the two groups. Consumers’ willingness to support exceeded businesses’ expectations for all support actions evaluated.

### 4.5. Consumers’ Transparency Perceptions of Safety Measures in Pandemic Times

#### 4.5.1. Communication by Food Service Businesses (Study 2)

Transparency perceptions regarding businesses’ communication of safety measures were calculated as a mean score of the eight items (McDonald’s omega = 0.940). No significant differences in perceived transparency were found for gender and education level, neither was there a correlation with age. However, perceived transparency was highly positively correlated with perceived compliance and perceived safety (Table 7), which were also strongly, and positively correlated. The more transparently businesses communicate, the more customers believe that businesses adhere to the imposed safety measures and the more they felt safe during their visit.

#### 4.5.2. Communication by the Government (Study 3)

Consumers’ transparency perceptions regarding government communications were measured for both lockdown periods. PCA was conducted on all 16 items and a factorial structure with two factors was recognized, i.e., eight items per lockdown. McDonald’s omega values justified the creation of composite measures. Table 8 summarizes the results. A significant increase in perceived transparency was observed with the progression of the pandemic (paired samples *t*-test; t=5.79; p<0.001), although consumers considered the government’s transparency for both lockdown periods to be fairly neutral.

Perceived transparency was also found to be positively correlated with support for the government’s decision to close food service businesses. The correlation was stronger for perceived transparency related to the communication in the second lockdown (r=0.623; p<0.001) compared to the first lockdown (r=0.392; p<0.001).

## 5. Discussion

By using the COVID-19 pandemic as a case, this study addresses the need for research on consumers’ changed behaviour regarding out-of-home food consumption during and following a pandemic. Based on three online surveys with 1697 consumers and 780 food service businesses, this study analysed (1) attitudes, intentions and behaviour regarding safety measures and dining out in pandemic times, and (2) transparency perceptions of safety measures. This research contributes to the current body of literature on out-of-home food consumption during the COVID-19 pandemic by integrating the perspectives of two stakeholder groups, i.e., food service consumers and businesses, and analysing their perceptions at different moments in time. Our findings provide important insights that will enable food service businesses to better understand consumers’ perceptions so they can anticipate them and ensure their own survival. The discussion is structured according to the research questions posed.

### 5.1. Consumers’ and Businesses’ Attitudes towards Safety Measures (RQ1a, RQ1b)

Our results indicate that, although consumers were generally concerned about all safety measures, attitudes towards them can be categorized into three factors related to hygiene, avoidance of object sharing and organisation. For both expected and imposed safety measures, sanitary measures, which focus on disinfection of hands and surfaces, are prioritized. This highlights the importance consumers attach to disinfecting when it comes to preventing virus transmission while consuming food out-of-home. The priority given to hygiene measures is similar to the results of previous consumer studies, where availability of disinfectants, staff wearing masks, extensive cleaning of surfaces, strict handwashing and training employees about sanitary practices were considered the most important precautions to be taken by restaurants [31,61]. However, food service businesses themselves did not expect their customers to attribute that much importance to the safety measures in place at their establishment. In addition, the profitability of restaurants and bars was severely compromised by the safety measures imposed.

### 5.2. Determinants of Consumers’ Revisit Intentions and Behaviour (RQ2)

Further, this study identifies different determinants of consumers’ intention and behaviour related to visiting food service businesses post-lockdown. When comparing consumers’ intentional and actual visiting behaviour, several differences can be recognized. While consumers’ attitudes towards all measures (hygiene, avoidance, organisation) had significant effects on the intention to revisit, only their attitudes towards hygiene measures were a significant factor influencing the likelihood to actually revisit and attitudes towards organisational measures were not that influential. In sum, the higher the importance attributed to (hygiene) measures, the less likely consumers were to (intend to) revisit. As such, it indicates that good compliance with (hygiene) measures appears to be an important strategy for businesses to regain customers when reopening is allowed. These findings are in line with previous studies that indicate the importance of cleanliness and sanitation when selecting and visiting restaurants [38,39] and confirm past studies in this field that identified consumers’ attitudes towards hygiene as a determinant of (re)visit intention [36,37]. Moreover, consumers’ cleanliness concerns are even heightened in times of global health crises, such as the current pandemic [30,32]. Our results underpin the importance of cleanliness and safety measures to draw customers back in by meeting their expectations [30]. Similar, cleanliness was used as selling proposition to recover from SARS [40]. However, Wei, et al. [61] observed different results and suggested that perceived importance of preventive COVID-19 measures indirectly enhanced customers’ intentions to dine out during the reopening period, through brand trust, i.e., customers’ reliance on a certain business. Implementing safety measures helps restaurants to build brand trust, even more for those who perceive high risk of COVID-19, and more trustworthy restaurants attract more customers [61,62]. Customers with a low risk perception of COVID-19 are less willing to adapt their lifestyle to comply with safety measures, hence their trust in restaurants is less impacted by the adoption of preventive measures [62]. Despite the seemingly contradictory results, the findings are similar to ours: by implementing and strictly complying with safety measures, restaurants might convince customers to resume dining out during the pandemic.

In addition, our results indicate that men state that they are more likely to revisit immediately while women state that they are more likely to postpone. This might be explained by gender differences in health-protective behavioural response to a respiratory pandemic [63]. Women are more concerned about COVID-19 and therefore take more precautions to avoid contamination [64], even though the severity and mortality are higher for male COVID-19 patients [65]. However, when it comes to actual revisit behaviour, gender is eventually not a determinant in our study, while age and educational level are. Being older as well as having achieved a higher level of education significantly decreases the likelihood of revisiting immediately. The age effect might be linked to a higher probability of severe illness for older people [65]. In a study by Hakim, et al. [34], though age did not impact visit intention, older customers’ visit intentions were less affected by denial of COVID-19 compared to younger people. Regarding educational status, Byrd, et al. [66] found that higher educated consumers have more concerns about the risk of contracting COVID-19 from restaurant food, which might explain their postponing behaviour, despite eating out-of-home more often [27]. Consumers’ pre-pandemic frequency of out-of-home consumption had a significant positive effect on the likelihood to intend to revisit and actually revisit. The more often people went out to eat or drink before COVID-19, the more likely they are to (plan to) do so again. Current results are consistent with Lee, et al. [67], who showed that the frequency of past travel behaviour was positively associated with the intention to travel during the 2009 H1N1 outbreak, and Mehrolia, et al. [26], who indicated that the probability of ordering food though online food delivery services during the COVID-19 lockdown was higher for customers with a higher purchasing frequency before.

### 5.3. Post-Pandemic Behaviour and Willingness to Support (RQ3a, RQ3b)

Our results indicate that consumers’ ordering frequency of takeaway meals doubled during mandatory closure periods of restaurants and bars, findings that are in line with Poelman, et al. [23]. Once the pandemic is over, consumers expect to return to their initial frequency of dining out and ordering takeaway.

Further, not only is the Belgian federal government financially helping food service businesses to overcome the current crisis, but consumers are also very willing to make personal contributions to support the sector, even more than was expected by restaurants and bars. Consumers indicate that they are highly willing to financially support to help food service businesses to survive, contrary to the expectations of the food service sector. Previous studies argued that high levels of solidarity with the food service sector have a positive effect on consumers’ intention to visit restaurants and bars [34] and to continue using food delivery applications [54] during the COVID-19 pandemic; however, this situation-specific effect is expected to diminish over time. The notable discrepancy that was found between consumers’ solidarity intentions and businesses’ expectations provides knowledge and opportunities for businesses to facilitate their survival of the pandemic.

### 5.4. Consumers’ Transparency Perceptions of Safety Measures in Pandemic Times (RQ4)

Perceived transparency of businesses’ communications about the imposed measures is positively correlated with perceived compliance of businesses with those measures and perceived safety of customers during their visit. The correlations between these variables suggest the importance of transparent communication to appear well-compliant and to make customers feel safe during their visit. These findings are in line with previous research, which argues that food service businesses can restore customers’ trust and encourage them to dine out during the pandemic by communicating in a transparent way [34,53].

Perceived transparency of government communication is positively correlated with the support for the mandatory closure decision. Similar results were observed by Scholz, et al. [47], who identified a positive correlation between the comprehensibility of a certain COVID-19-related decision, in particular of its underlying rationale, and its acceptance. Providing timely, clear, and consistent policy recommendations improves compliance [46,49]. As consumers’ perceptions of transparency increased throughout the pandemic, this might have positive implications for the acceptance of more recent government decisions.

## 6. Conclusions

### 6.1. Implications

This study contributes to the growing body of COVID-19-related literature in the hospitality domain. It is one of the first studies to assess the role of safety measures in predicting consumers’ revisit intention and behaviour. By demonstrating the importance consumers attribute to safety measures and hygiene when resuming visits to restaurants and bars, this study helps to better understand consumers’ preferences regarding out-of-home food consumption during a pandemic. Further, while previous research has mostly focused on either consumers or food service businesses at one moment in time, this study extends the existing literature by integrating perspectives of two key stakeholder groups at multiple stages of the pandemic.

The COVID-19 pandemic has tremendously impacted food service business operations. The findings of this study can help the food service sector in developing adequate survival strategies. As both consumers’ revisit intention and behaviour were determined by their attitudes towards sanitary measures, this study highlights how adoption of and adherence to safety measures may be an effective approach for food service businesses to attract customers in pandemic times. Further, it is suggested that by communicating transparently about these measures, business owners will make their customers feel safe during their visits. This study also revealed consumers’ high willingness to contribute financially to the continued existence of restaurants and bars. The food service sector should benefit from these solidarity intentions as they are likely to decline over time. Understanding customers’ expectations and willingness to provide support, both in terms of financial contributions during lockdown periods and physical visits during reopening periods, might help business owners face the challenges posed by this and future health crises.

### 6.2. Limitations and Future Research

The present study has some limitations. Our results are based on data from Flanders, Belgium, collected during the first and second wave of COVID-19. Perceptions and attitudes may differ from country to country, as the COVID-19 pandemic has affected countries in various ways and to various extents and has been tackled by various policy decisions. Moreover, although the pandemic is still ongoing, perceptions and attitudes may change over time as both consumers and food service businesses are gradually adjusting to the new normal. Future research could investigate the long-term effects of COVID-19 on out-of-home food consumption behaviour, both in later stages of the pandemic and when the pandemic would be over. Furthermore, caution is needed when interpreting consumers’ views on visit intention and solidarity actions as they may deviate from actual behaviour, known as the intention–behaviour gap [68]. Finally, future research could further expand the variables used in this study. Besides attitudes towards safety measures and past behaviour, consumers’ revisit intentions and behaviours could be affected by other factors, e.g., risk perception related to COVID-19 infection could also be relevant. To further explore the role of perceived transparency regarding communication in pandemic times, its effects on trust and compliance could be investigated. Future studies may also deepen the understanding of the discrepancies found between consumers’ attitudes and businesses’ expectations and elaborate on consumers’ changed consumption behaviour.

## Figures and Tables

**Table 1:** Overview of survey development and data collection for the three studies.

	Study 1	Study 2	Study 3
**Survey Development**			
	**Consumers**	**Consumers**	**Consumers**
	(*n* = 1083)	(*n* = 309)	(*n* = 305)
Part 1: Behavioural variables	Visit frequency before COVID-19	Visit frequency before COVID-19	Takeaway frequency before and since COVID-19
	Revisit intention	Revisit behaviour	
Part 2: Attitudes and perceptions	Attitudes towards 21 expected safety measures	Attitudes towards 14 imposed safety measures	Attitudes towards government decisions
		Perceived safety and compliance	Willingness to support through 5 actions
		Perceived business transparency of safety measures	Perceived government transparency of measures
Part 3: Profiling variables	Socio-demographic	Socio-demographic	Socio-demographic
	**Food service sector**	**Food service sector**	**Food service sector**
	(*n* = 306)	(*n* = 221)	(*n* = 253)
Part 1: Attitudes and perceptions	Expectations of attitudes towards 21 expected safety measures	Perceived impact on profitability of 14 imposed safety measures	Expectations of willingness to support through 5 actions
Part 2: Profiling variables	Business type	Business type	Business type
**Data Collection**			
Timing	May 2020	June 2020	November 2020
Stage of the pandemic	1st wave of infections	In between waves	2nd wave of infections
Situation for food service businesses (start date)	Mandatory closure (14 March 2020)	Reopening (8 June 2020)	Mandatory closure (19 October 2020)

**Table 2:** Socio-demographic characteristics of the sample per study.

	Study 1	Study 2	Study 3
**Consumers**	(*n* = 1083) (%)	(*n* = 309) (%)	(*n* = 305) (%)
*Age*			
Mean (SD)	42.40 (13.73)	43.99 (14.66)	43.98 (15.10)
*Gender*			
Male	38.1	36.9	43.6
Female	61.9	63.1	56.4
*Education*			
Primary or secondary	29.8	16.5	21.6
Higher	70.2	83.5	78.4
**Food service sector**	(*n* = 306) (%)	(*n* = 221) (%)	(*n* = 253) (%)
*Business type*			
Restaurant (serving food and drinks)	81.0	78.7	73.5
Bar (only serving drinks)	19.0	21.3	26.5

**Table 3:** Factor loadings from principal component analysis for consumers’ attitudes towards expected safety measures (study 1; *n* = 1083).

Items	Mean	S.D.	Factor 1	Factor 2	Factor 3
Disinfectants available on the table	3.42	1.20	**0.729**	−0.002	0.157
Staff disinfects toilet after each visit	3.92	1.13	**0.645**	0.293	0.111
Staff disinfects hands after clearing each table	4.21	0.98	**0.640**	0.324	0.114
Service is performed with mouth mask	3.62	1.22	**0.638**	0.257	0.300
Service is provided with gloves	3.11	1.34	**0.631**	0.101	0.203
Tables and chairs are disinfected after each visit	4.06	1.03	**0.598**	0.385	0.248
Mandatory disinfection of hands upon arrival	4.41	0.87	**0.500**	0.299	0.266
Newspapers and magazines are not provided	3.80	1.23	0.204	**0.799**	0.201
No possibility of self-service or buffet	3.99	1.14	0.174	**0.736**	0.247
Menus and drinks menus are not interchangeable between tables	3.97	1.04	0.312	**0.720**	0.225
Clients must hang their own coat in the checkroom	3.39	1.10	0.095	**0.538**	0.268
Only disposable consumables on the table	3.33	1.36	0.404	**0.536**	0.119
Mandatory reservation by clients	3.16	1.39	0.019	0.215	**0.700**
Customers received in shifts per time block	3.11	1.22	0.247	0.108	**0.681**
Seating only under guidance	3.69	1.26	0.196	0.244	**0.673**
Presence of walking paths	3.17	1.26	0.399	0.112	**0.609**
Clients can only consume while seated	3.58	1.23	0.206	0.395	**0.556**
Availability of waiting zones upon arrival	3.45	1.10	0.302	0.301	**0.536**
McDonald’s omega			0.827	0.812	0.799
Mean (S.D.)			3.82 (0.78)	3.70 (0.88)	3.36 (0.88)

Note: KMO measure of sampling adequacy: 0.939; Bartlett’s test of sphericity: 7445.125 (*p* < 0.001); bold indicates on which factor an item loads highest (loading > 0.5).

**Table 4:** Coefficient estimates and diagnostics from hierarchical binary logistic regression explaining consumers’ revisit intentions (study 1; *n* = 1053).

	Model 1: Consumer Profiling Variables					Model 2: Consumer Profiling and Attitudes				
Variable	B	S.E.	Wald	*p*	Exp(B)	B	S.E.	Wald	*p*	Exp(B)
*Socio-demographic*										
Age	−0.001	0.005	0.039	0.843	0.999	0.009	0.005	3.099	0.078	1.009
Gender (1 = male)	0.488	0.142	11.910	**0.001**	1.630	0.204	0.154	1.760	0.185	1.226
Education (1 = higher)	−0.141	0.151	0.872	0.350	0.868	−0.159	0.163	0.958	0.328	0.853
*Past behaviour*										
Visit frequency	0.335	0.049	47.305	<**0.001**	1.398	0.300	0.050	36.555	<**0.001**	1.349
*Attitudes*										
F1(1) Hygiene						−0.479	0.138	12.043	**0.001**	0.620
F2(1) Avoidance						−0.449	0.121	13.727	<**0.001**	0.638
F3(1) Organisation						−0.505	0.122	17.197	<**0.001**	0.604
Constant	−0.256	0.276	0.862	0.353	0.774	4.849	0.578	70.427	<**0.001**	127.613
*Model*										
Likelihood ratio	101.971			<0.001		244.007			<0.001	
Nagelkerke R^2^	0.125					0.279				

Note: Predictive accuracy of 63.6% (Model 1) and 69.3% (Model 2) compared to 59.8% in the ‘null’ model; dependent variable (revisit intention) is a dummy variable: postpone visit (0), retake visit (1); bold indicates significant coefficients (*p* < 0.05); FX(Y) with X = number of factor, Y = number of study.

**Table 5:** Factor loadings from principal component analysis for consumers’ attitudes towards imposed safety measures (study 2; *n* = 309 ).

Items	Mean	S.D.	Factor 1	Factor 2
Tables and chairs are disinfected after each visit	4.26	0.96	**0.794**	0.227
Only paper towels and lockable bins in the toilets	4.47	0.76	**0.769**	0.176
Payment terminal is disinfected after each use or hand gels/cotton buds available	4.17	1.00	**0.756**	0.270
Disinfectants available for clients	4.38	0.79	**0.745**	0.208
Service is performed with mouth mask	4.02	1.10	**0.686**	0.462
Kitchen staff wears mouth mask or keeps distance	4.06	1.11	**0.668**	0.363
Glasses are washed with soap	4.50	0.74	**0.654**	0.240
Mandatory closure at 1 am	3.00	1.38	0.117	**0.801**
Clients can only consume while seated	3.69	1.22	0.332	**0.787**
Maximum of 10 clients per table	3.76	1.14	0.272	**0.748**
Distance of 1.5 m is maintained outside and inside	4.21	0.96	0.422	**0.587**
McDonald’s omega			0.892	0.805
Mean (S.D.)			4.27 (0.72)	3.66 (0.93)

Note: KMO measure of sampling adequacy: 0.896; Bartlett’s test of sphericity: 1778.693 (*p* < 0.001); bold indicates on which factor an item loads highest (loading > 0.5).

**Table 6:** Coefficient estimates and diagnostics from hierarchical binary logistic regression explaining consumers’ revisit behaviour (study 2; *n* = 301 ).

	Model 1: Consumer Profiling Variables					Model 2: Consumer Profiling and Attitudes				
Variable	B	S.E.	Wald	*p*	Exp(B)	B	S.E.	Wald	*p*	Exp(B)
*Socio-demographic*										
Age	−0.028	0.010	7.509	**0.006**	0.972	−0.022	0.011	4.097	**0.043**	0.978
Gender (1 = male)	0.608	0.313	3.771	0.052	1.837	0.465	0.324	2.057	0.151	1.591
Education (1 = higher)	−1.023	0.467	4.797	**0.029**	0.360	−1.034	0.482	4.602	**0.032**	0.356
*Past behaviour*										
Visit frequency	0.965	0.197	24.060	<0.001	2.626	0.968	0.210	21.245	<**0.001**	2.632
*Attitudes*										
F1(2) Hygiene						−0.830	0.329	6.371	**0.012**	0.436
F2(2) Organisation						−0.170	0.233	0.531	0.466	0.844
Constant	1.664	0.750	4.918	**0.027**	5.279	5.743	1.392	17.023	<**0.001**	311.952
*Model*										
Likelihood ratio	68.029			<0.001		84.051			<0.001	
Nagelkerke R^2^	0.289					0.348				

Note: Predictive accuracy of 75.1% (Model 1) and 76.1% (Model 2) compared to 71.1% in the ‘null’ model; dependent variable (revisit behaviour) is a dummy variable: visit postponed (0), visit retaken (1); bold indicates significant coefficients (*p* < 0.05); FX(Y) with X = number of factor, Y = number of study.

**Table 7:** Bivariate correlations between consumers’ perceived transparency, compliance, and safety (study 2; n=214).

	Mean	S.D.	Perceived Transparency	Perceived Compliance
Perceived transparency	3.92	0.84	1	
Perceived compliance	4.05	1.04	0.596 ***	1
Perceived safety	4.18	0.95	0.602 ***	0.785 ***

Note: *** p<0.001.

**Table 8:** Factor loadings from principal component analysis for perceived transparency (study 3; n=305).

Items	1st Lockdown	2nd Lockdown
	Factor 1	Factor 2
Information is timely	0.700	0.634
Information is relevant	0.763	0.793
Information is consistent	0.795	0.692
Information is complete	0.786	0.799
Information is easy to understand	0.806	0.774
Information is accurate	0.843	0.784
Information is reliable	0.769	0.829
Information explains the rationale	0.716	0.745
McDonald’s omega	0.917	0.909
Mean (S.D.)	2.86 (0.88)	3.16 (0.88)

Note: KMO measure of adequacy: 0.910; Bartlett’s test of sphericity: 3225.558 (p<0.001); only factor loadings above 0.5 are presented.

## Data Availability

Data will be made available on request.
